# 3,6,14,17-Tetramethoxy-22,23-diphenyl-1,10,12,21-tetraazahexacyclo[19.2.1.0^2,7^.0^10,23^.0^12,22^.0^13,18^]tetracosa-2(7),3,5,13(18),14,16-hexaene-11,24-di­thione

**DOI:** 10.1107/S160053681002204X

**Published:** 2010-06-16

**Authors:** Yan Yang

**Affiliations:** a Key Laboratory of Pesticides and Chemical Biology of Ministry of Education, College of Chemistry, Central China Normal University, Wuhan 430079, People’s Republic of China

## Abstract

The title compound, C_36_H_34_N_4_O_4_S_2_, is a thio­glycoluril derivative, which bears two phenyl substituents on its convex face and two meth­oxy substituted *o*-xylylenes as sidewalls of the molecular clip. There is one half-mol­ecule in the asymmetric unit: a crystallographic twofold axis generates the complete molecule. The non-planar seven-membered rings adopt chair conformations, while the two five-membered rings exhibit envelope conformations and make a dihedral angle of 68.46 (12)°. The O atoms of the meth­oxy groups are coplanar with the six-membered *o*-xylylene sidewalls.

## Related literature

For related structures, see: Broan *et al.* (1989 ▶); Cao *et al.* (2009 ▶); Wang *et al.* (2006 ▶); Wang & Xi (2009 ▶); Wu & Sun, (2009 ▶). For further synthetic details, see: Broan *et al.* (1989 ▶); Wu *et al.* (2002 ▶). The rigid concave shape of glycoluril makes it a versatile building block in supramolecular chemistry, see: Gao *et al.* (2009 ▶); Rowan *et al.* (1999 ▶); Hof *et al.* (2002 ▶); Kolbel & Menger (2001 ▶); Wu *et al.* (2002 ▶); Kang *et al.* (2004 ▶).
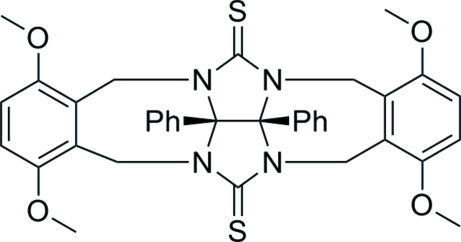


## Experimental

### 

#### Crystal data

C_36_H_34_N_4_O_4_S_2_
*M* *_r_* = 650.79
Monoclinic, 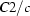

*a* = 17.9993 (15) Å
*b* = 12.5069 (11) Å
*c* = 16.0934 (12) Å
β = 115.961 (3)°
*V* = 3257.3 (5) Å^3^
*Z* = 4
Mo *K*α radiation
μ = 0.21 mm^−1^
*T* = 298 K
0.23 × 0.20 × 0.10 mm

#### Data collection

Bruker SMART CCD area-detector diffractometer
13570 measured reflections
3546 independent reflections
2279 reflections with *I* > 2σ(*I*)
*R*_int_ = 0.067

#### Refinement

*R*[*F* ^2^ > 2σ(*F* ^2^)] = 0.056
*wR*(*F* ^2^) = 0.162
*S* = 0.98
3546 reflections
210 parameters
H-atom parameters constrained
Δρ_max_ = 0.29 e Å^−3^
Δρ_min_ = −0.24 e Å^−3^

### 

Data collection: *SMART* (Bruker, 2001 ▶); cell refinement: *SAINT-Plus* (Bruker, 2001 ▶); data reduction: *SAINT-Plus*; program(s) used to solve structure: *SHELXS97* (Sheldrick, 2008 ▶); program(s) used to refine structure: *SHELXL97* (Sheldrick, 2008 ▶); molecular graphics: *PLATON* (Spek, 2009 ▶); software used to prepare material for publication: *PLATON*.

## Supplementary Material

Crystal structure: contains datablocks I, global. DOI: 10.1107/S160053681002204X/fl2304sup1.cif

Structure factors: contains datablocks I. DOI: 10.1107/S160053681002204X/fl2304Isup2.hkl

Additional supplementary materials:  crystallographic information; 3D view; checkCIF report

## Figures and Tables

**Fig. 1.**
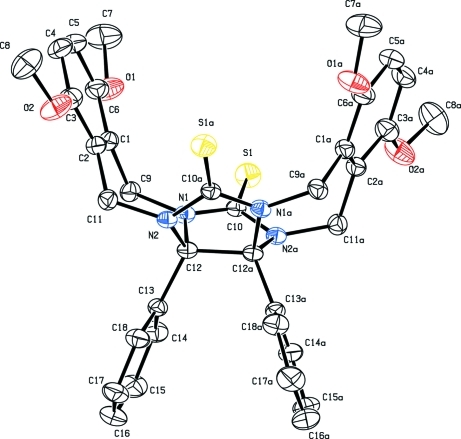
A view of (I), showing the atom-labelling scheme, with displacement ellipsoids drawn at the 30% probability level.

**Fig. 2.**
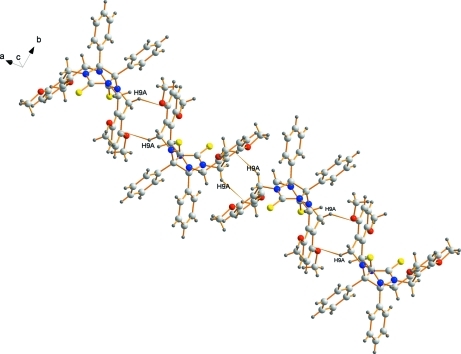
Packing of (I) with C—H···π interactions drawn as dashed lines showing the formation of a one-dimensional chain.

## supplementary crystallographic information

### Comment

The rigid concave shape of glycoluril makes it a versatile building block to construct various supramolecular objects (Gao *et al.*, 2009), including molecular clips and molecular baskets (Rowan *et al.*, 1999), molecular capsules (Hof *et al.*, 2002), xerogels (Kolbel & Menger, 2001), the cucurbit[n]uril family (Wu *et al.*, 2002), and anion-binding receptors (Kang *et al.*, 2004). Based on the previous studies (Broan *et al.*, 1989; Cao *et al.*, 2009; Wang *et al.*, 2006; Wang & Xi, 2009; Wu & Sun, 2009), we report here the structure of the title thioglycoluril derivative (Fig. 1), which is a potential receptor in supramolecular chemistry.

There is one half-molecule in the asymmetric unit. The non-planar seven-membered rings adopt chair conformations, while the two five-membered rings have envelope conformation and the dihedral angle between them is 68.46°. The methoxy groups on sidewalls are coplanar with the six-membered o-xylylene sidewalls. The molecule contains three nonclassical intramolecular C—H···S, C—H···O and C—H···N hydrogen bonds, and its crystal structure is stabilized mostly by intermolecular C—H···π interactions (Table 1).

### Experimental

The thioglycoluril was synthesized according to a literature procedure, see : Broan *et al.*, (1989). Preparation of the title compound: A solution of thioglycoluril (326 mg, 1.00 mmol), paraformaldehyde (120 mg, 4.00 mmol) and 1,4-dimethoxybenzene (304 mg, 2.20 mmol) in TFA (5 ml) was stirred and heated at reflux for 6 h. After rotary evaporation the residue was chromatographed to yield the tile compound (521 mg, 0.80 mmol,80%). Crystals of (I) suitable for X-ray diffraction were grown by slow evaporation of a dichloromethane-methanol (1:2) solution of the title compound under 293 K.

### Refinement

All H atoms were positioned in geometrically idealized positions and constrained to ride on their parent atoms, with C—H distances in the range 0.93-0.98 Å and Uĩso~(H) = 1.2U~eq~(C) or Uĩso~(H) = 1.5U~eq~(C).

### Crystal data

**Table:**

C_36_H_34_N_4_O_4_S_2_	*F*(000) = 1368
*M**_r_* = 650.79	*D*_x_ = 1.327 Mg m^−^^3^
Monoclinic, *C*2/*c*	Mo *K*α radiation, λ = 0.71073 Å
Hall symbol: -C 2yc	Cell parameters from 2697 reflections
*a* = 17.9993 (15) Å	θ = 2.5–21.3°
*b* = 12.5069 (11) Å	µ = 0.21 mm^−^^1^
*c* = 16.0934 (12) Å	*T* = 298 K
β = 115.961 (3)°	Block, colorless
*V* = 3257.3 (5) Å^3^	0.23 × 0.20 × 0.10 mm
*Z* = 4	

### Data collection

**Table:**

Bruker SMART CCD area-detector diffractometer	2279 reflections with *I* > 2σ(*I*)
Radiation source: fine-focus sealed tube	*R*_int_ = 0.067
graphite	θ_max_ = 27.0°, θ_min_ = 2.1°
phi and ω scans	*h* = −22→13
13570 measured reflections	*k* = −15→15
3546 independent reflections	*l* = −19→20

### Refinement

**Table:**

Refinement on *F*^2^	Primary atom site location: structure-invariant direct methods
Least-squares matrix: full	Secondary atom site location: difference Fourier map
*R*[*F*^2^ > 2σ(*F*^2^)] = 0.056	Hydrogen site location: inferred from neighbouring sites
*wR*(*F*^2^) = 0.162	H-atom parameters constrained
*S* = 0.98	*w* = 1/[σ^2^(*F*_o_^2^) + (0.0878*P*)^2^] where *P* = (*F*_o_^2^ + 2*F*_c_^2^)/3
3546 reflections	(Δ/σ)_max_ = 0.001
210 parameters	Δρ_max_ = 0.29 e Å^−^^3^
0 restraints	Δρ_min_ = −0.24 e Å^−^^3^

### Special details

**Table:**

Geometry. All esds (except the esd in the dihedral angle between two l.s. planes) are estimated using the full covariance matrix. The cell esds are taken into account individually in the estimation of esds in distances, angles and torsion angles; correlations between esds in cell parameters are only used when they are defined by crystal symmetry. An approximate (isotropic) treatment of cell esds is used for estimating esds involving l.s. planes.
Refinement. Refinement of *F*^2^ against ALL reflections. The weighted *R*-factor wR and goodness of fit *S* are based on *F*^2^, conventional *R*-factors *R* are based on *F*, with *F* set to zero for negative *F*^2^. The threshold expression of *F*^2^ > σ(*F*^2^) is used only for calculating *R*-factors(gt) etc. and is not relevant to the choice of reflections for refinement. *R*-factors based on *F*^2^ are statistically about twice as large as those based on *F*, and *R*- factors based on ALL data will be even larger.

### Fractional atomic coordinates and isotropic or equivalent isotropic displacement parameters (Å^2^)

**Table:**

	*x*	*y*	*z*	*U*_iso_*/*U*_eq_	
S1	0.11451 (4)	0.22118 (5)	0.14339 (5)	0.0528 (3)	
N1	0.09215 (11)	0.31734 (13)	0.27979 (13)	0.0335 (5)	
N2	−0.00738 (11)	0.34780 (13)	0.14082 (13)	0.0325 (4)	
C13	0.07957 (13)	0.50126 (16)	0.32973 (15)	0.0343 (5)	
C9	0.16813 (14)	0.27755 (17)	0.35521 (17)	0.0388 (6)	
H9A	0.1970	0.3371	0.3949	0.047*	
H9B	0.2036	0.2489	0.3294	0.047*	
C12	0.03855 (13)	0.39284 (16)	0.29759 (15)	0.0306 (5)	
C10	0.06573 (14)	0.29489 (16)	0.18917 (16)	0.0326 (5)	
O2	0.06197 (14)	0.16264 (16)	0.57355 (15)	0.0713 (6)	
O1	0.24132 (13)	0.08009 (14)	0.37734 (15)	0.0683 (6)	
C1	0.15415 (14)	0.19204 (17)	0.41322 (17)	0.0393 (6)	
C2	0.10798 (15)	0.21274 (17)	0.46227 (17)	0.0406 (6)	
C6	0.19350 (16)	0.09225 (18)	0.42349 (19)	0.0477 (7)	
C18	0.13789 (15)	0.53676 (19)	0.30210 (19)	0.0497 (7)	
H18	0.1560	0.4916	0.2688	0.060*	
C11	−0.06462 (15)	0.31850 (18)	0.04628 (16)	0.0392 (6)	
H11A	−0.0343	0.3157	0.0092	0.047*	
H11B	−0.1060	0.3742	0.0209	0.047*	
C3	0.10368 (17)	0.1344 (2)	0.52320 (19)	0.0512 (7)	
C14	0.05429 (16)	0.56979 (18)	0.37978 (18)	0.0463 (7)	
H14	0.0150	0.5469	0.3988	0.056*	
C4	0.14195 (17)	0.0364 (2)	0.5309 (2)	0.0578 (8)	
H4	0.1379	−0.0156	0.5701	0.069*	
C17	0.16966 (18)	0.6394 (2)	0.3237 (2)	0.0655 (9)	
H17	0.2086	0.6631	0.3045	0.079*	
C5	0.18588 (18)	0.0156 (2)	0.4811 (2)	0.0564 (8)	
H5	0.2108	−0.0508	0.4863	0.068*	
C15	0.0865 (2)	0.6717 (2)	0.4019 (2)	0.0638 (9)	
H15	0.0693	0.7170	0.4360	0.077*	
C16	0.1438 (2)	0.7055 (2)	0.3733 (2)	0.0731 (10)	
H16	0.1654	0.7742	0.3878	0.088*	
C7	0.2729 (2)	−0.0224 (2)	0.3750 (3)	0.0859 (11)	
H7A	0.2924	−0.0550	0.4348	0.129*	
H7B	0.3178	−0.0162	0.3583	0.129*	
H7C	0.2301	−0.0658	0.3302	0.129*	
C8	0.0599 (2)	0.0907 (3)	0.6401 (2)	0.0865 (11)	
H8A	0.0241	0.0319	0.6094	0.130*	
H8B	0.0396	0.1269	0.6787	0.130*	
H8C	0.1146	0.0643	0.6775	0.130*	

### Atomic displacement parameters (Å^2^)

**Table:**

	*U*^11^	*U*^22^	*U*^33^	*U*^12^	*U*^13^	*U*^23^
S1	0.0520 (5)	0.0520 (4)	0.0612 (5)	0.0130 (3)	0.0311 (4)	−0.0081 (3)
N1	0.0300 (11)	0.0293 (9)	0.0435 (12)	0.0043 (7)	0.0181 (9)	0.0014 (8)
N2	0.0341 (11)	0.0273 (9)	0.0390 (11)	0.0020 (8)	0.0189 (9)	−0.0016 (8)
C13	0.0316 (13)	0.0300 (11)	0.0397 (13)	−0.0029 (9)	0.0141 (10)	0.0006 (9)
C9	0.0268 (13)	0.0385 (13)	0.0466 (14)	0.0035 (10)	0.0118 (11)	0.0042 (10)
C12	0.0317 (12)	0.0264 (10)	0.0391 (12)	0.0013 (9)	0.0205 (10)	0.0006 (9)
C10	0.0327 (13)	0.0270 (11)	0.0395 (14)	−0.0012 (9)	0.0170 (11)	0.0011 (9)
O2	0.0922 (16)	0.0662 (13)	0.0746 (14)	0.0174 (11)	0.0543 (13)	0.0311 (11)
O1	0.0756 (15)	0.0481 (11)	0.0968 (17)	0.0248 (10)	0.0521 (13)	0.0162 (10)
C1	0.0328 (14)	0.0343 (12)	0.0433 (14)	0.0000 (10)	0.0097 (11)	0.0030 (10)
C2	0.0390 (14)	0.0331 (12)	0.0430 (14)	−0.0015 (10)	0.0118 (12)	0.0037 (10)
C6	0.0452 (16)	0.0364 (13)	0.0582 (17)	0.0053 (11)	0.0197 (13)	0.0014 (11)
C18	0.0428 (16)	0.0428 (14)	0.0692 (19)	−0.0048 (11)	0.0300 (14)	0.0017 (12)
C11	0.0397 (14)	0.0408 (13)	0.0357 (13)	0.0020 (10)	0.0153 (11)	−0.0017 (10)
C3	0.0549 (18)	0.0474 (15)	0.0536 (17)	−0.0009 (13)	0.0259 (14)	0.0083 (12)
C14	0.0582 (17)	0.0334 (13)	0.0518 (16)	−0.0035 (11)	0.0284 (14)	−0.0037 (11)
C4	0.063 (2)	0.0437 (15)	0.0596 (18)	0.0013 (13)	0.0207 (16)	0.0190 (13)
C17	0.0486 (18)	0.0531 (17)	0.091 (2)	−0.0183 (14)	0.0267 (17)	0.0117 (16)
C5	0.065 (2)	0.0345 (14)	0.0639 (19)	0.0073 (12)	0.0226 (16)	0.0073 (13)
C15	0.090 (2)	0.0359 (14)	0.0624 (19)	−0.0065 (15)	0.0304 (17)	−0.0115 (13)
C16	0.084 (3)	0.0387 (16)	0.080 (2)	−0.0232 (16)	0.021 (2)	−0.0042 (15)
C7	0.112 (3)	0.055 (2)	0.113 (3)	0.0199 (19)	0.070 (3)	−0.0035 (18)
C8	0.099 (3)	0.095 (3)	0.076 (2)	0.009 (2)	0.048 (2)	0.036 (2)

### Geometric parameters (Å, °)

**Table:**

S1—C10	1.652 (2)	C6—C5	1.381 (4)
N1—C10	1.351 (3)	C18—C17	1.386 (4)
N1—C9	1.462 (3)	C18—H18	0.9300
N1—C12	1.465 (3)	C11—C2^i^	1.511 (3)
N2—C10	1.371 (3)	C11—H11A	0.9700
N2—C12^i^	1.450 (3)	C11—H11B	0.9700
N2—C11	1.462 (3)	C3—C4	1.384 (4)
C13—C18	1.381 (3)	C14—C15	1.381 (3)
C13—C14	1.382 (3)	C14—H14	0.9300
C13—C12	1.522 (3)	C4—C5	1.375 (4)
C9—C1	1.511 (3)	C4—H4	0.9300
C9—H9A	0.9700	C17—C16	1.364 (4)
C9—H9B	0.9700	C17—H17	0.9300
C12—N2^i^	1.450 (3)	C5—H5	0.9300
C12—C12^i^	1.553 (4)	C15—C16	1.369 (4)
O2—C3	1.370 (3)	C15—H15	0.9300
O2—C8	1.412 (3)	C16—H16	0.9300
O1—C6	1.369 (3)	C7—H7A	0.9600
O1—C7	1.410 (3)	C7—H7B	0.9600
C1—C2	1.398 (3)	C7—H7C	0.9600
C1—C6	1.409 (3)	C8—H8A	0.9600
C2—C3	1.412 (3)	C8—H8B	0.9600
C2—C11^i^	1.511 (3)	C8—H8C	0.9600
			
C10—N1—C9	125.79 (19)	N2—C11—H11A	108.6
C10—N1—C12	113.23 (17)	C2^i^—C11—H11A	108.6
C9—N1—C12	120.91 (18)	N2—C11—H11B	108.6
C10—N2—C12^i^	111.24 (18)	C2^i^—C11—H11B	108.6
C10—N2—C11	122.26 (18)	H11A—C11—H11B	107.6
C12^i^—N2—C11	120.09 (18)	O2—C3—C4	123.7 (2)
C18—C13—C14	118.6 (2)	O2—C3—C2	116.3 (2)
C18—C13—C12	120.1 (2)	C4—C3—C2	120.0 (3)
C14—C13—C12	121.1 (2)	C15—C14—C13	121.0 (3)
N1—C9—C1	113.92 (19)	C15—C14—H14	119.5
N1—C9—H9A	108.8	C13—C14—H14	119.5
C1—C9—H9A	108.8	C5—C4—C3	120.4 (2)
N1—C9—H9B	108.8	C5—C4—H4	119.8
C1—C9—H9B	108.8	C3—C4—H4	119.8
H9A—C9—H9B	107.7	C16—C17—C18	120.0 (3)
N2^i^—C12—N1	111.61 (16)	C16—C17—H17	120.0
N2^i^—C12—C13	112.90 (18)	C18—C17—H17	120.0
N1—C12—C13	112.20 (18)	C4—C5—C6	120.8 (2)
N2^i^—C12—C12^i^	103.2 (2)	C4—C5—H5	119.6
N1—C12—C12^i^	100.82 (17)	C6—C5—H5	119.6
C13—C12—C12^i^	115.23 (12)	C16—C15—C14	119.4 (3)
N1—C10—N2	108.02 (18)	C16—C15—H15	120.3
N1—C10—S1	126.35 (17)	C14—C15—H15	120.3
N2—C10—S1	125.57 (17)	C17—C16—C15	120.7 (3)
C3—O2—C8	119.2 (2)	C17—C16—H16	119.7
C6—O1—C7	118.2 (2)	C15—C16—H16	119.7
C2—C1—C6	119.4 (2)	O1—C7—H7A	109.5
C2—C1—C9	121.2 (2)	O1—C7—H7B	109.5
C6—C1—C9	119.2 (2)	H7A—C7—H7B	109.5
C1—C2—C3	119.4 (2)	O1—C7—H7C	109.5
C1—C2—C11^i^	121.4 (2)	H7A—C7—H7C	109.5
C3—C2—C11^i^	119.2 (2)	H7B—C7—H7C	109.5
O1—C6—C5	123.9 (2)	O2—C8—H8A	109.5
O1—C6—C1	116.0 (2)	O2—C8—H8B	109.5
C5—C6—C1	120.0 (3)	H8A—C8—H8B	109.5
C13—C18—C17	120.3 (3)	O2—C8—H8C	109.5
C13—C18—H18	119.8	H8A—C8—H8C	109.5
C17—C18—H18	119.8	H8B—C8—H8C	109.5
N2—C11—C2^i^	114.50 (19)		
			
C10—N1—C9—C1	−106.3 (3)	C7—O1—C6—C5	11.7 (4)
C12—N1—C9—C1	77.1 (3)	C7—O1—C6—C1	−171.6 (3)
C10—N1—C12—N2^i^	122.1 (2)	C2—C1—C6—O1	−176.5 (2)
C9—N1—C12—N2^i^	−60.9 (2)	C9—C1—C6—O1	−1.2 (3)
C10—N1—C12—C13	−110.1 (2)	C2—C1—C6—C5	0.4 (4)
C9—N1—C12—C13	66.9 (2)	C9—C1—C6—C5	175.7 (2)
C10—N1—C12—C12^i^	13.1 (2)	C14—C13—C18—C17	−0.5 (4)
C9—N1—C12—C12^i^	−169.92 (18)	C12—C13—C18—C17	173.8 (2)
C18—C13—C12—N2^i^	155.7 (2)	C10—N2—C11—C2^i^	70.0 (3)
C14—C13—C12—N2^i^	−30.1 (3)	C12^i^—N2—C11—C2^i^	−79.4 (2)
C18—C13—C12—N1	28.6 (3)	C8—O2—C3—C4	2.6 (4)
C14—C13—C12—N1	−157.3 (2)	C8—O2—C3—C2	−175.9 (3)
C18—C13—C12—C12^i^	−86.0 (3)	C1—C2—C3—O2	175.8 (2)
C14—C13—C12—C12^i^	88.1 (3)	C11^i^—C2—C3—O2	−2.3 (4)
C9—N1—C10—N2	−179.57 (19)	C1—C2—C3—C4	−2.8 (4)
C12—N1—C10—N2	−2.7 (2)	C11^i^—C2—C3—C4	179.0 (2)
C9—N1—C10—S1	−2.1 (3)	C18—C13—C14—C15	0.0 (4)
C12—N1—C10—S1	174.75 (16)	C12—C13—C14—C15	−174.3 (2)
C12^i^—N2—C10—N1	−10.2 (2)	O2—C3—C4—C5	−177.0 (3)
C11—N2—C10—N1	−162.05 (19)	C2—C3—C4—C5	1.5 (4)
C12^i^—N2—C10—S1	172.29 (15)	C13—C18—C17—C16	0.6 (4)
C11—N2—C10—S1	20.5 (3)	C3—C4—C5—C6	0.8 (4)
N1—C9—C1—C2	−61.3 (3)	O1—C6—C5—C4	174.9 (2)
N1—C9—C1—C6	123.5 (2)	C1—C6—C5—C4	−1.8 (4)
C6—C1—C2—C3	1.8 (4)	C13—C14—C15—C16	0.5 (4)
C9—C1—C2—C3	−173.4 (2)	C18—C17—C16—C15	−0.2 (5)
C6—C1—C2—C11^i^	180.0 (2)	C14—C15—C16—C17	−0.4 (5)
C9—C1—C2—C11^i^	4.8 (3)		

Symmetry codes: (i) −*x*, *y*, −*z*+1/2.

### Hydrogen-bond geometry (Å, °)

**Table:**

Cg1 is the centroid of the C1–C6 ring.

**Table:**

*D*—H···*A*	*D*—H	H···*A*	*D*···*A*	*D*—H···*A*
C9—H9A···Cg1^ii^	0.97	2.70	3.540	145
C11—H11A···O2^ii^	0.97	2.26	2.757 (3)	111
C14—H14···N2^ii^	0.93	2.56	2.879 (3)	101
C18—H18···N1	0.93	2.51	2.842 (3)	102
C9—H9B···S1	0.97	2.73	3.189 (3)	110
C9—H9B···O1	0.97	2.25	2.748 (3)	111

Symmetry codes: (ii) −*x*, *y*, −*z*+1/2.
